# Measured multi-source semi-supervised working condition recognition based on curvelet pooling and attention mechanism learning

**DOI:** 10.1038/s41598-025-23732-2

**Published:** 2025-11-14

**Authors:** Shuo Yang, Bin Zhou, Yanjiang Wang, Weifeng Liu

**Affiliations:** 1 School of Computer Science and Technology, Shandong University of Technology, Zibo, 255000 People’s Republic of China; 2 College of Control Science and Engineering, China University of Petroleum (East China), Qingdao, 266580 People’s Republic of China

**Keywords:** Sucker-rod pumping well working condition recognition, Multi-source feature fusion, Semi-supervised learning, Curvelet pooling, Attention mechanism, Energy science and technology, Engineering, Mathematics and computing

## Abstract

To identify various oil well working conditions more accurately and practically from massive image data collected by multiple measured information sources of sucker-rod pumping wells, this paper proposes a working condition recognition method with three key aspects: curvelet pooling optimization technology, multi-source attention mechanism fusion feature extraction technology, and multi-source semi-supervised classification deep learning. Specifically: (a) Curvelet pooling optimization technology. We introduce the second-generation curvelet transform into the ResNet-50 pooling layer and adopt a collaborative learning pooling strategy of low-frequency and high-frequency information from the raw data decomposed via curvelet transform instead of max-pooling. This enhances the neural network’s capability to capture detailed features of complex image data. (b) Multi-source attention mechanism fusion feature extraction technology. We selected two information sources: measured ground dynamometer cards and measured electrical power cards. The multi-head self-attention mechanism enables interactive complementarity between curvelet-decomposed image data from each information source, while achieving dynamic weighted fusion of the interactive complementary data via the adaptive attention mechanism. This process yields optimal global feature representations of multi-source fused data. (c) Multi-source semi-supervised classification deep learning. By integrating multi-source fused feature data with a semi-supervised classification algorithm based on the dual strategy of dynamic adjustment of pseudo-label confidence and self-adaptive class fairness regularization, the method leverages abundant multi-source unlabeled samples to improve model classification performance and generalization ability under limited labeled training samples. This further enhances the accuracy and practicality of condition recognition. Experimental data were collected from a high-pressure, low-permeability, thin oil reservoir block in an oilfield in China. Extensive experiments demonstrate that the proposed method efficiently processes measured information source data in the sucker-rod pumping production system, improves the performance of traditional deep learning frameworks, explores the intrinsic correlations among multiple measured information source data of oil wells, and utilizes massive unlabeled working condition data to enhance the working condition recognition effect and engineering practicability with a minimal number of labeled samples. Code is available at https://github.com/Yoick/AMMFFECP.

## Introduction

In the context of the Internet of Things for oil and gas production, the sucker-rod pumping well production system can acquire massive measured working condition data, derived from multiple information sources such as ground dynamometer cards, electrical parameters et al., and also obtain a large amount of unlabeled working condition data. These data from diverse measured information sources are characterized by strong noise, high dimensionality, and significant randomness, yet they realistically and comprehensively reflect the operational status of pumping wells. Traditional signal processing techniques can no longer effectively handle such high-dimensional and complex data^1,2^. Deep learning technology excels at extracting deep feature representations from high-dimensional complex data by constructing machine learning models with multiple hidden layers, thereby enhancing model classification or prediction performance. However, the requirement for a large number of labeled training samples^3^ and the neglect of valuable unlabeled samples^4^ have constrained the engineering application of deep learning. The sucker-rod pumping well production system is a complex nonlinear system with the mechanic-electronic-liquid coupling, making it impossible for single-source information or traditional intelligent information processing techniques to efficiently identify working conditions in an accurate manner^5,6^. Thus, exploring how to effectively utilize multiple measured information sources at the pumping well site, leverage deep learning technology, and integrate massive unlabeled samples with a minimal number of existing labeled samples to enhance the performance and practicality of working condition recognition models holds significant theory and engineering value.

Currently, most deep learning-based working condition recognition methods for sucker-rod pumping wells rely on convolutional neural network architectures^7^. While these methods have demonstrated promising results, they suffer from a significant limitation: the loss of local key information during signal processing^8^. Specifically, classical CNNs typically employ max-pooling or average pooling to reduce the data dimensionality^9^. This technique overlooks the subtle and multi-scale characteristics of complex signals, inevitably leading to the loss of local key information within the working condition data. To address this issue, researchers in related fields have proposed integrating multi-scale transformation techniques into the CNN pooling layer to replace traditional pooling methods. Wavelet transform, renowned for its superior multi-scale decomposition capabilities, has been widely adopted in pooling layer enhancements. By preserving image details, reducing noise, and improving the model’s ability to capture multi-scale features, wavelet transform offers notable improvements^10,11^. However, it exhibits limitations when handling directional and complex edge features. With its low directional resolution, wavelet transform can only decompose signals in horizontal, vertical, and diagonal directions, rendering it ineffective in capturing curved edges and intricate curve structures in images. This shortcoming is particularly pronounced in tasks requiring precise multi-directional and detailed information extraction. In contrast, the curvelet transform^12,13^ not only inherits multi-scale properties but also enables multi-directional decomposition, allowing for more accurate capture of curve and edge information in images. The curvelet pooling technique, grounded in curvelet transform^14^, demonstrates significant advantages in retaining complex structural features and fine details of images, making it highly suitable for image processing tasks with stringent geometric feature requirements. Therefore, incorporating curvelet pooling into CNN-based working condition recognition methods can effectively mitigate the loss of local feature information in working condition data.

Most of the existing research on the working condition recognition of sucker-rod pumping wells primarily relies on single-source data analysis, such as dynamometer cards^15^ or electrical parameters^16^. However, these methods often yield inaccurate results when applied to complex and dynamic oil well conditions, failing to meet the urgent demand for efficient and precise identification in the context of accelerating oilfield digitalization. To address this challenge, recent studies have shifted focus toward multi-source fusion technologies, aiming to leverage complementary advantages among diverse data sources to enhance recognition performance. For example, researchers have combined dynamometer cards, electrical parameters, and oil production statistics through multi-feature concatenation^17,18^. While these approaches mitigate the limitations of single-source analysis, they introduce new challenges: (a) Robustness Issues. Uncertainties in production statistics can compromise model stability. (b) Inefficient Feature Fusion. The multi-feature concatenation struggles to capture deep inter-source correlations and often introduces redundant information under high-dimensional conditions, degrading both efficiency and accuracy. Weighted fusion methods^19^ attempt to improve integration by assigning fixed weights to different sources. However, these weights are typically determined via prior knowledge and fail to adapt dynamically to complex data relationships, limiting model generalization. In contrast, attention mechanisms in deep learning offer two key advantages: (a) Latent Relationship Capture: By enabling interactive learning across information sources, attention mechanisms uncover hidden correlations^20^. (b) Dynamic Weight Adjustment: They dynamically prioritize relevant features, enhancing model performance^21^. Thus, integrating attention mechanisms can effectively overcome the limitations of current multi-source fusion approaches for working condition recognition.

The requirement for a large number of labeled working condition samples has severely restricted the engineering application of existing sucker-rod pumping well working condition recognition technologies^22^. To address this issue, some scholars have proposed using a small amount of labeled dynamometer card data combined with meta-learning methods for the working condition recognition^4^, while others have suggested leveraging a small amount of labeled dynamometer card data and a large amount of unlabeled dynamometer card data in conjunction with semi-supervised generative adversarial networks^23^. Although these studies have effectively tackled the problem of requiring a large number of labeled training samples, their engineering practicality is limited by the constraints of single-source recognition and the weak generalization ability of the models.

In the field of semi-supervised deep learning, pseudo-label learning offers high flexibility and strong generalization capabilities, making it suitable for various practical application scenarios. Combining pseudo-label learning with consistency regularization can further enhance the performance of semi-supervised learning models^24,25^. Pseudo-label learning based on a dynamic confidence adjustment strategy can efficiently filter out low-quality pseudo-labels, while self-adaptive class fairness regularization learning can dynamically balance the prediction distributions of different categories. Integrating these two techniques with multi-source feature fusion technology can effectively overcome the limitations of single-source-based semi-supervised deep learning for the working condition recognition in the complex sucker-rod pumping well system, thereby improving the generalization performance and robustness of working condition recognition models in semi-supervised scenarios.

In summary, this paper proposes a measured multi-source semi-supervised working condition recognition method based on curvelet pooling and attention mechanism learning, addressing the limitations of existing sucker-rod pumping well working condition recognition research while integrating the characteristics of big data oil production. Specifically, we first introduce the second-generation curvelet transform into the ResNet-50 pooling layer, replacing max-pooling with a collaborative learning pooling strategy that utilizes low-frequency and high-frequency information from curvelet-decomposed raw data. We then select two information sources: measured ground dynamometer cards and measured electrical power cards, and employ a multi-head self-attention mechanism to enable interactive complementarity between curvelet-decomposed image data of each source. An adaptive attention mechanism further achieves a dynamic weighted fusion of the interactive complementary data, thereby obtaining the optimal global feature representation of multi-source fused data. Finally, we integrate the multi-source fused feature data with a semi-supervised classification algorithm based on a dual strategy of pseudo-label confidence dynamic adjustment and self-adaptive class fairness regularization. This approach leverages limited multi-source labeled training samples alongside abundant unlabeled samples to enhance model classification performance and generalization ability, ultimately improving the accuracy and practicality of working condition recognition.

The contributions of this paper are as follows:

- We propose an efficient curvelet pooling optimization technology. Based on the ResNet-50 architecture, we introduce the second-generation curvelet transform into the ResNet-50 pooling layer, replacing the original max-pooling with a collaborative learning pooling strategy that utilizes low-frequency and high-frequency information from curvelet-decomposed raw data. This enhancement enables the neural network to capture detailed features of complex image data, overcoming the limitation of local key information loss in traditional CNNs during signal processing;
- We propose an attention mechanism-based multi-source fusion feature extraction technology. By selecting two information sources: measured ground dynamometer cards and measured electrical power cards, we employ a multi-head self-attention mechanism to enable interactive learning across sources, effectively capturing latent relationships between multi-source features. An adaptive attention mechanism then achieves dynamic weighted fusion across sources, yielding optimal global feature representations of multi-source fused data;
- We propose an efficient multi-source semi-supervised deep learning method for working condition recognition. This method integrates the curvelet pooling optimization and attention-based multi-source feature fusion with a semi-supervised classification algorithm featuring dynamic pseudo-label confidence adjustment and self-adaptive class fairness regularization. The integration allows effective utilization of limited labeled data and abundant unlabeled data to enhance recognition accuracy and practicality;
- Extensive experiments validate the proposed method’s effectiveness. Compared with traditional single/multi-source approaches, different pooling techniques, and semi-supervised methods under varying labeling ratios, the proposed method demonstrates superior performance—especially with only 10% labeled data. Hyperparameter sensitivity and ablation studies further confirm its robustness.

## Related work

At present, the working condition recognition technologies for sucker-rod pumping wells can be categorized into three types: dynamometer card-based methods, electrical parameter-based methods, and multi-source data-based methods.

### Research status of dynamometer card-based working condition recognition

Traditional dynamometer card-based techniques construct classification models by integrating specific feature extraction strategies with machine learning methods to achieve accurate recognition of working conditions. For example, Wu et al.^26^ proposed a three-level wavelet packet decomposition method, which normalized dynamometer card data, extracted feature vectors, and classified working conditions using an RBF neural network. Li et al.^27^ partitioned dynamometer cards via the ’four-point method’, extracted moment-invariant features, and employed a PSO-optimized SVM for recognition. Zhang and Gao^28^ utilized the fast discrete curvelet transform for feature extraction and a sparse multi-graph regularized extreme learning machine for classification.

Although these traditional methods have achieved moderate success, they suffer from three key limitations: reliance on manual feature design, insufficient generalization capability, and the need for large labeled samples. Deep learning technologies based on convolutional neural networks (CNNs) have emerged as a research focus due to their powerful feature extraction capabilities. Wang et al.^29^ proposed using CNNs to extract dynamometer card features, enabling intelligent diagnosis of pumping well conditions without manual intervention or prior knowledge, but this approach relied heavily on abundant labeled samples. Zhang et al.^30^ applied transfer learning with CNNs to significantly improve the working condition diagnosis performance on small-sample datasets and alleviated the generalization limitations of shallow networks. He et al.^4^ developed a working condition recognition method based on 4-dimensional time-frequency signatures and a meta-learning convolutional shrinkage neural network, enhancing recognition accuracy through time-frequency feature extraction and low-frequency feature ablation while addressing few-shot problems via a meta-learning framework.

These methods still require performance improvements and overlook the value of abundant unlabeled samples. Additionally, to address the demand for large labeled samples, Moussa et al.^23^ proposed semi-supervised generative adversarial networks, which generated synthetic dynamometer cards by combining labeled and unlabeled data to enable the working condition recognition with minimal labeled samples. However, classification accuracy under low labeling ratios remains suboptimal. Notable Limitations: (a) Single-source vulnerability. Sole reliance on dynamometer cards causes misdiagnosis for complex conditions. (b) Labeling bottleneck. Most methods require a plentiful supply of labeled samples, unfitting to the actual engineering requirements. (c) CNN feature degradation. Standard pooling loses local critical features for subtle fault detection.

### Research status of electrical parameter-based working condition recognition

Electrical parameters have garnered sustained attention due to their easy acquisition and cost-effectiveness. Zheng et al.^31^ proposed extracting seven novel features from electrical power data, combining key parameters such as valve opening/closing points and pump working cycles, and using multiple hidden conditional random fields for the working condition recognition. Chen et al.^16^ extracted 16 time-domain and frequency-domain features from power curves based on the working mechanism of sucker-rod pumps, employing XGBoost for classification. Tecle et al.^32^ generated motor power curves considering subsurface condition influences through a sucker-rod pump simulation model, using these curves with SVM for classification.

While these studies have achieved certain results, traditional feature extraction and machine learning methods show inadequacies when facing massive data. Thus, research on electrical parameter-based working condition recognition has gradually shifted to deep learning approaches. Wei and Gao^33^ proposed using CNN to extract electrical power features, fusing them with manually engineered features via mechanical analysis, and then using a generalized learning system as a classifier for the working condition recognition. These electrical parameter-based methods have made progress, but still have limitations: the “division by zero” issue, which originates from the up-and-down reciprocating mechanical motion characteristics of the sucker-rod pumping system’s four-bar linkage. Specifically, the torque coefficient is zero at the upper and lower dead centers of the suspension point of the polish rod. When it is used as a divisor, a large error will occur, thus affecting the calculation precision of the feature parameters; single-source data is prone to misjudgments; the demand for large labeled samples hinders engineering applications; and CNN-based methods suffer from key information loss.

### Research status of multi-source data-based working condition recognition

The complex field environment makes single-source data insufficient to comprehensively represent pumping well conditions, driving multi-source working condition recognition to become an important research direction. Liu et al.^34^ proposed AdaBNet (Adaptive Weighted Ensemble Bayesian Network) for fault monitoring by integrating features such as dynamometer card area, maximum/minimum load, daily operation time, and production statistics. Zhang et al.^35^ established a comprehensive diagnostic model based on feature recognition by extracting features from dynamometer cards and power vs. position plots under different working conditions. Xu et al.^36^ proposed a machine learning diagnostic model fusing electrical parameters and dynamometer cards, setting multi-stage constraints and optimizing parameters via an adaptive genetic algorithm for precise condition assessment.

The above methods fail to fully exploit the potentiality among information sources, still relying on manually designed feature extraction. Traditional multi-feature concatenation has reached a bottleneck, and the use of statistical features weakens model robustness, limiting adaptability under complex conditions. Zhou et al.^37^ developed a multi-source p-Laplacian regularized kernel logistic regression model using dynamometer cards, electrical power signals, and wellhead temperature signals for measured multi-source semi-supervised working condition recognition, but its performance in multi-classification needs improvement.

To more efficiently utilize multi-source information, researchers have explored deep learning-based methods. Li et al.^38^ fused Fourier descriptor and graphical features of dynamometer cards to exploit complementary advantages, enhancing the diagnostic model performance. Abdurakipov et al.^18^ built a working condition recognition model by integrating dynamometer cards, pump inlet pressure and temperature data with Transformer. Although these deep learning methods have achieved results, they still underutilize latent correlations between multi-source features and perform poorly with few labeled samples, restricting practical application.

Based on the above analysis, CNN-based single-source deep learning methods have shown significant effectiveness, and extending them to multi-source learning is expected to improve the model performance. However, the inherent pooling design of traditional CNNs limits multi-source recognition. While wavelet pooling^39^ alleviates this issue, it struggles with images rich in edge details and texture features. By contrast, curvelet pooling^14^ more effectively mitigates feature loss caused by traditional CNN pooling. Additionally, existing multi-source learning methods rely on manual weight setting for information sources, weakening synergy. Li et al.^40^ proposed an adaptive data fusion strategy using deep learning, implementing fault diagnosis via adaptive convolution kernels matching multi-source channels. Liu et al.^21^ developed an attention-based adaptive multi-scale module to dynamically adjust feature weights across sources, providing references for pumping well multi-source learning. Meanwhile, working condition recognition based on multi-source learning is constrained by large labeled sample requirements. Semi-supervised techniques based on pseudo-label learning and consistency regularization have matured in semi-supervised deep learning, although lacking multi-source interactive learning, their semi-supervised concepts effectively address the over-reliance on labeled samples in current research.

## Methodology

### Problem definition

The key symbols used in this paper and their detailed descriptions are shown in Table 1.

**Table 1:** Notation description.

Notation	Description
*D*	Collection of multi-source datasets
*V*	Number of information sources
${L}^{v}$	Labeled dataset of the *v*-th information source
${U}^{v}$	Unlabeled dataset of the *v*-th information source
${x}_{i}^{v}$	The *i*-th sample of the *v*-th information source
${y}_{i}$	Label of the *i*-th sample in each source
*k*	Total number of labeled samples in each source
*m*	Total number of unlabeled samples in each source
*C*	Total number of categories in the dataset
$C\left( I\right)$	Curvelet pooling layer
*T*	Confidence threshold for pseudo-labeling
$\lambda$	Momentum decay coefficient of EMA
$\mu$	The ratio of unlabeled to labeled data batch sizes
${L}_{s}$	Supervised learning loss
${L}_{c}$	Multi-source feature interaction constraint loss
${L}_{u}$	Unsupervised learning loss
${L}_{f}$	Self-adaptive class fairness regularization loss
${{w}}_{c}$	Weight of multi-source feature interaction constraint loss
${{w}}_{u}$	Weight of unsupervised learning loss
${{w}}_{f}$	Weight of self-adaptive class fairness regularization loss

This paper proposes a multi-source semi-supervised working condition recognition method for sucker-rod pumping wells based on curvelet pooling and attention mechanism learning. We construct a multi-source dataset *D* containing *V* different information sources, where each information source contains *C* categories. Dataset *D* is divided into two components: the labeled dataset *L* and the unlabeled dataset *U*, that is, $D=\{L,U\}$. The labeled dataset *L* is defined as $L=\{L^v\}=\{(x_i^v, y_i)\}_{i=1}^k$, where $L^v$ represents the labeled dataset of the *v*-th information source, $x_i^v$ is the *i*-th sample in the *v*-th information source, $y_i\in \{1,2,\ldots ,C\}$ is the sample label, and *k* is the total number of labeled samples. The unlabeled dataset *U* is defined as $U=\{U^v\}=\{x_j^v\}_{j=k+1}^{k+m}$, where $U^v$ is the unlabeled dataset of the *v*-th information source, $x_j^v$ is the *j*-th unlabeled sample from the *v*-th information source, and *m* is the total number of unlabeled samples.

### Model overview

**Fig. 1:**
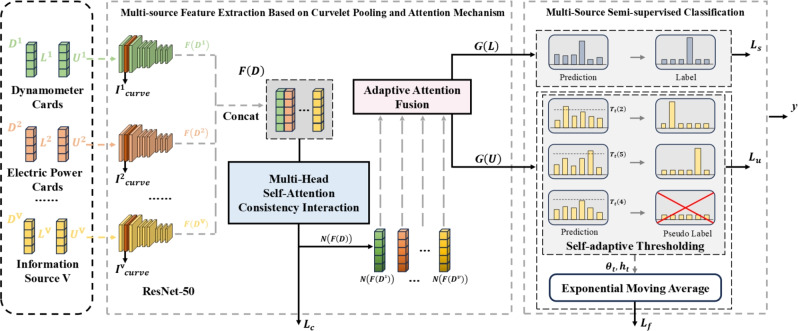
An overview of the model framework.

The model framework is illustrated in Fig. 1, primarily composed of two submodules. The multi-source feature extraction submodule based on curvelet pooling and attention mechanism learning first employs ResNet-50 as the basic architecture, introducing the curvelet transform to replace the max-pooling layers with curvelet pooling layers. This enhances the network’s capability to capture fine-grained features from complex images. The multi-head self-attention mechanism then processes multi-source feature representations in parallel, capturing cross-source dependencies across different subspaces to enable efficient feature interaction. A cosine similarity-based multi-source feature interaction constraint loss function is introduced to quantify feature similarity, further enhancing the cross-source interaction effect by maximizing this similarity. An adaptive attention mechanism finally learns weight coefficients for features from different sources, fusing multi-source representations to generate optimal global feature vectors for each sample. The multi-source semi-supervised classification submodule draws inspiration from the FreeMatch framework, leveraging abundant unlabeled samples in multi-source datasets under limited labeled conditions. It introduces self-adaptive confidence thresholding to dynamically filter high-quality pseudo-labels and self-adaptive class fairness regularization to balance class-wise prediction distributions. These mechanisms collectively improve the model’s generalization ability and classification performance in semi-supervised scenarios, effectively addressing the challenge of limited labeled data in practical applications.

#### A multi-source feature extraction submodule based on curvelet pooling and attention mechanism learning

##### Curvelet pooling

In the field of sucker-rod pumping well working condition recognition, signals such as dynamometer cards and electrical power cards exhibit rich texture and edge features, which are crucial for identifying operational states and detecting potential faults. However, traditional convolutional neural networks often weaken or lose key edge information when reducing the spatial dimension of feature maps via max-pooling layers, degrading overall model performance.

Compared with the wavelet transform, the curvelet transform preserves edge and texture information more effectively due to its multi-scale and multi-directional properties. This enables neural networks to learn richer feature representations, improving the model’s capability to recognize complex images. In this paper, ResNet-50 serves as the base network architecture, with traditional max-pooling layers replaced by curvelet pooling layers based on curvelet transform. This optimization enhances the network’s feature extraction efficiency, improves the model’s sensitivity to fine-grained features, and boosts performance in complex condition recognition tasks.

Specifically, for the input sample ${x}_{i}^{v}$, the curvelet pooling layer is defined in Eq. 1:

1 $$\begin{aligned} C\left( x_i^v\right) =C_J+\sum _{j=1}^J D_j \end{aligned}$$

where $C_J$ represents the low-frequency component, and $D_j$ represents the high-frequency component of different scales $j ( j =1,2,\ldots ,J )$. The low-frequency component $C_J$ and high-frequency component $D_j$ are aggregated through direct element-wise summation. This additive fusion method preserves both the global contextual information captured by $C_J$ and the discriminative multi-scale details retained in $D_j$. In terms of tensor dimensions, the input ${x}_{i}^{v}$ has a shape of $B$ $\times$ $C$ $\times$ $H$ $\times$ $W$, $C_J$ is of size $B$ $\times$ $C$ $\times$ $H_{J}$ $\times$ $W_{J}$, while each component $D_j$ at scale $j ( j =1,2,\ldots ,J )$ has dimensions $B$ $\times$ $C$ $\times$ $H_{j}$ $\times$ $W_{j}$. The resulting output $I$, obtained through element-wise aggregation of these components, has a shape of $B$ $\times$ $C$ $\times$ $H^{\prime }$ $\times$ $W^{\prime }$.

The curvelet pooling layer is integrated into the network to replace the max-pooling layer, as expressed in Eq. 2.

2 $$\begin{aligned} I_{\text{ curve } }^v=C\left( I_{\text{ conv } }^v\right) \end{aligned}$$

where $I_{\text{ conv } }^v$denotes the output of the convolutional layer in ResNet-50, and $I_{\text{ curve } }^v$ represents the output of the curvelet pooling.

**Fig. 2:**
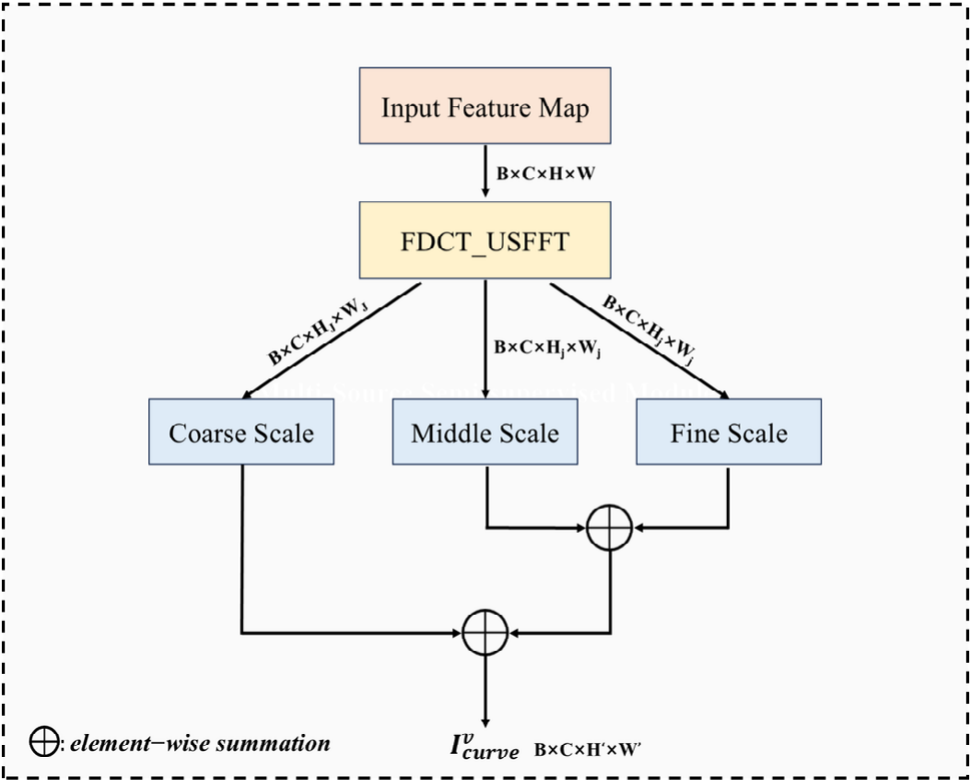
Structure diagram of the curvelet pooling layer based on ResNet-50.

The architecture of the curvelet pooling layer is illustrated in Fig. 2. In the diagram, FDCT-USFFT (fast discrete curvelet transforms based on unequally-spaced fast Fourier transform) represents the curvelet transform method based on USFFT, where ’Coarse Scale’ corresponds to the coarsest scale of low-frequency information after transformation, ’Fine Scale’ corresponds to the finest scale of high-frequency information, and ’Middle Scale’ represents other high-frequency information.

By incorporating the curvelet pooling layer, the enhanced ResNet-50 model can more effectively capture the key edge features of sucker-rod pumping well working condition samples. It guides the model training process by jointly leveraging low-frequency and high-frequency information, thereby improving the accuracy and robustness of working condition recognition.

##### Multi-source feature interaction learning based on multi-head self-attention mechanism

Inspired by the transformer^41^ coding architecture, this component employs a multi-head self-attention mechanism and feedforward neural network to process multi-source feature representations in parallel. It captures cross-source feature dependencies across different subspaces and performs nonlinear transformations to enable multi-source feature interaction. A cosine similarity-based multi-source feature interaction constraint loss function is introduced to ensure maximum consistency of multi-source feature representations in the feature space, thereby enhancing model generalization.

**Fig. 3:**
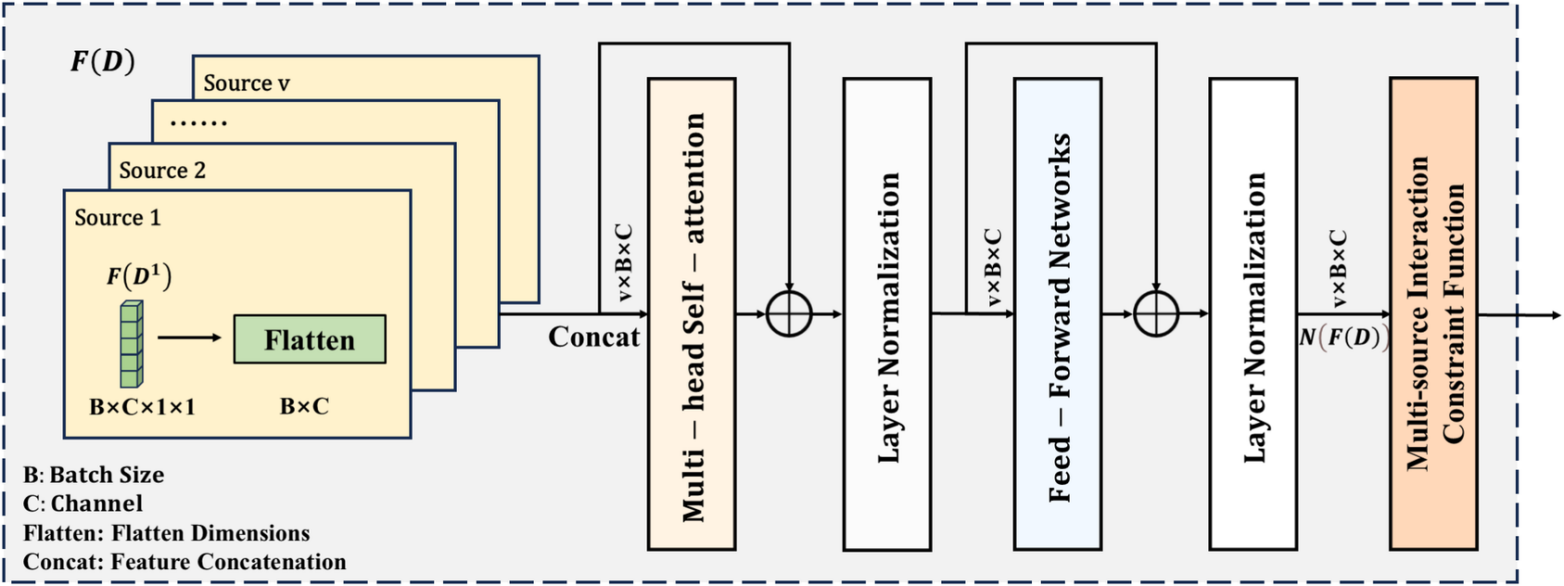
Schematic diagram of multi-source feature interaction learning based on multi-head self-attention mechanism.

The schematic diagram of multi-source feature interaction learning based on multi-head self-attention mechanism is shown in Fig. 3. First, multi-source features $F(D)$ outputted by curvelet pooling optimized ResNet-50 are flattened and concatenated, then fed into the multi-head self-attention module (*mhsa*). MHSA captures cross-source correlation information across different subspaces, effectively enhancing multi-source feature expressiveness. Training stability is maintained via residual connections and layer normalization. Next, the feedforward network (*ffn*) performs nonlinear transformations to extract deeper features, with residual connections and layer normalization again ensuring training stability, as defined in Eq. (3):

3 $$\begin{aligned} N(F(D))={\textit{ffn}}(m h s a[F(D)]) \end{aligned}$$

where $N(F(D))$ denotes multi-source features after interaction, and the *ffn* consists of two linear mapping layers with a ReLU activation function.

To fully exploit the intrinsic interactions between multi-source data and improve model generalization, a cosine similarity-based multi-source feature interaction constraint loss is proposed. This loss maximizes cosine similarity between cross-source feature representations to ensure their consistency in the feature space. Specifically, first, for the cross-source feature representations: (a) Normalize via $L_2$ norms to eliminate magnitude differences. (b) Calculate the cosine similarity of the *v*-th source feature, as in Eq. (4):

4 $$\begin{aligned} S^v=\frac{N\left( F\left( D^v\right) \right) \cdot N\left( F\left( D^v\right) \right) ^T}{\left\| N\left( F\left( D^v\right) \right) \right\| _2 \cdot \left\| N\left( F\left( D^v\right) \right) ^T\right\| _2} \end{aligned}$$

where $S^v$ denotes the cosine similarity of the *v*-th source feature, ${N\left( F\left( D^v\right) \right) }$ denotes the feature representation of the *v*-th source after the multi-source interaction, and $(\cdot )^T$ denotes vector transposition.

Then, compute the difference matrix between any two source similarity matrices, and the multi-source feature interaction constraint loss ${L}_{c}$ is defined as the square of the norm of the difference matrix, ensuring cross-source feature consistency in the feature space, as shown in Eq. (5):

5 $$\begin{aligned} L_c=\sum _{i, j \in V}\left\| S^i-S^j\right\| _2^2 \end{aligned}$$

##### Multi-source feature fusion based on adaptive attention mechanism

**Fig. 4:**
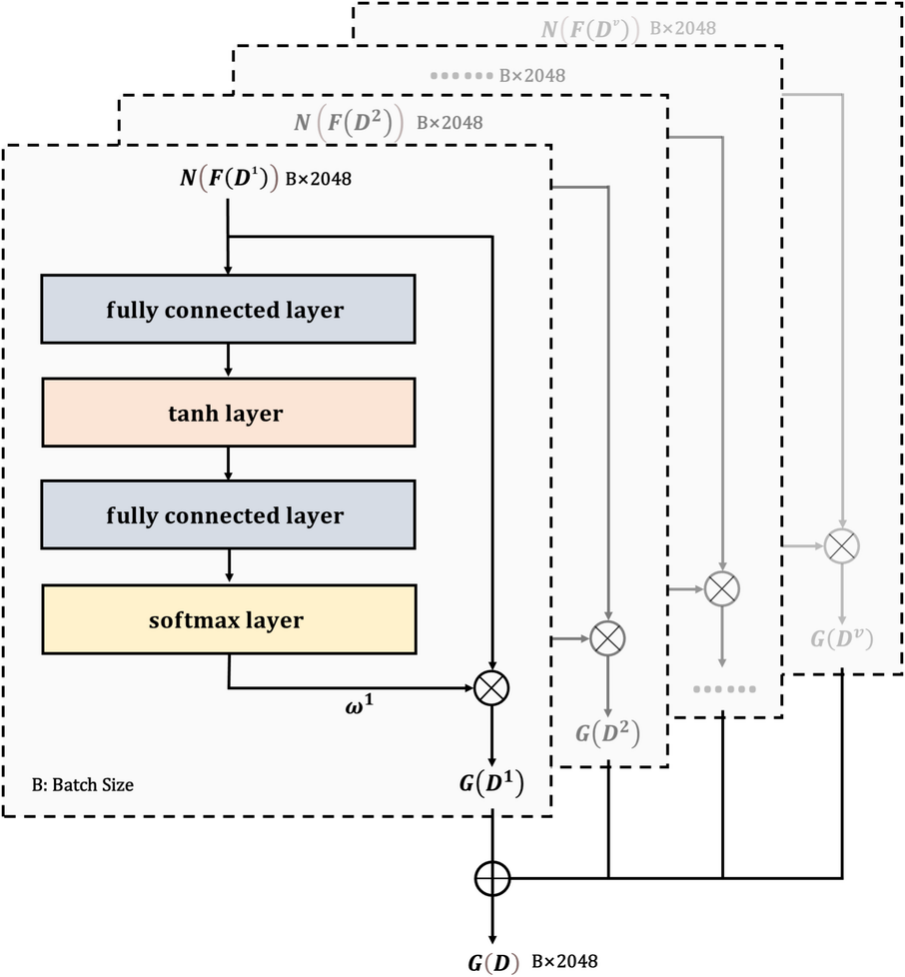
Schematic diagram of multi-source feature fusion based on adaptive attention mechanism.

This component introduces an adaptive attention mechanism to dynamically learn representation weights from different source features. By assigning differentiated weights, the model enhances its capability to capture key information, enabling effective fusion of interacted multi-source feature representations. The mechanism consists of the fully connected layer, the tanh activation layer, the fully connected layer, and the softmax layer in sequence, as illustrated in Fig. 4.

For the feature representation $N\left( F\left( {D}^{v}\right) \right)$ of the *v*-th source data, the process unfolds as follows: first, the feature is transformed via the fully connected layer, followed by non-linear mapping through the tanh activation function. The second fully connected layer and softmax function then compute the attention score $\omega ^v$. The calculated weight $\omega ^v$ is multiplied by $N(F(D^v))$ to weight the feature, and finally, weighted feature representations from all sources are fused to obtain the global feature representation, as shown in Eq. (6):

6 $$\begin{aligned} G(D)=\sum _{v=1}^V \omega ^v \cdot N\left( F\left( D^v\right) \right) \end{aligned}$$

where $G(D)$ represents the fused global feature representation, which provides a richer feature representation for subsequent classification

#### Multi-source semi-supervised classification submodule

This submodule performs semi-supervised multi-source classification on the global feature representation $G(D)$. Inspired by the FreeMatch algorithm, it optimizes model training under limited labeled samples by introducing a self-adaptive confidence threshold and self-adaptive class fairness regularization for the multi-source feature representation $G(U)$ of unlabeled samples. These mechanisms enhance the classification performance and generalization ability of the model.

The self-adaptive confidence threshold dynamically adjusts based on the model’s prediction confidence for unlabeled samples. A lower threshold in the early training phase enables full utilization of unlabeled data, while the threshold adjusts synchronously as the model confidence improves to suppress the negative impact of false labeling. The global threshold $T_t$ is updated via exponential moving average (EMA), as shown in Eq. (7):

7 $$\begin{aligned} T_t=\lambda T_{t-1}+(1-\lambda ) \frac{\sum _{b=1}^{\mu B} \max \left( q_b\right) }{\mu B} \end{aligned}$$

where $\lambda$ is the EMA momentum decay coefficient, $B$ is the batch size, and ${q}_{b}$ is the model’s prediction confidence for unlabeled samples.

The update of the local threshold ${\tilde{\theta }}_t(c)$ is shown in Eq. (8):

8 $$\begin{aligned} {\tilde{\theta }}_t(c)= {\left\{ \begin{array}{ll}\frac{1}{c}, & t=0 \\ (1-\lambda ) \frac{1}{\mu B} \sum _{b=1}^{\mu B} q_b(c)+\lambda {\tilde{\theta }}_{t-1}(c), & { otherwise }\end{array}\right. } \end{aligned}$$

where *c* represents the category corresponding to the current threshold, and $q_b(c)$ represents the confidence level predicted for that category. This threshold is independently and dynamically adjusted based on the predicted confidence distribution of each category, thereby effectively suppressing noise interference in categories with high uncertainty during the early training stage and progressively refining the screening precision in later phases. This mechanism effectively mitigates the issue of pseudo-label quality degradation.

The self-adaptive confidence threshold at different times can be calculated based on the dynamic update formula of the global threshold and the local threshold, as shown in Eq. (9):

9 $$\begin{aligned} T_t(c)=\frac{{\tilde{\theta }}_t(c)}{\max \left\{ {\tilde{\theta }}_t(c): c \in [c]\right\} } \cdot T_t \end{aligned}$$

**Fig. 5:**
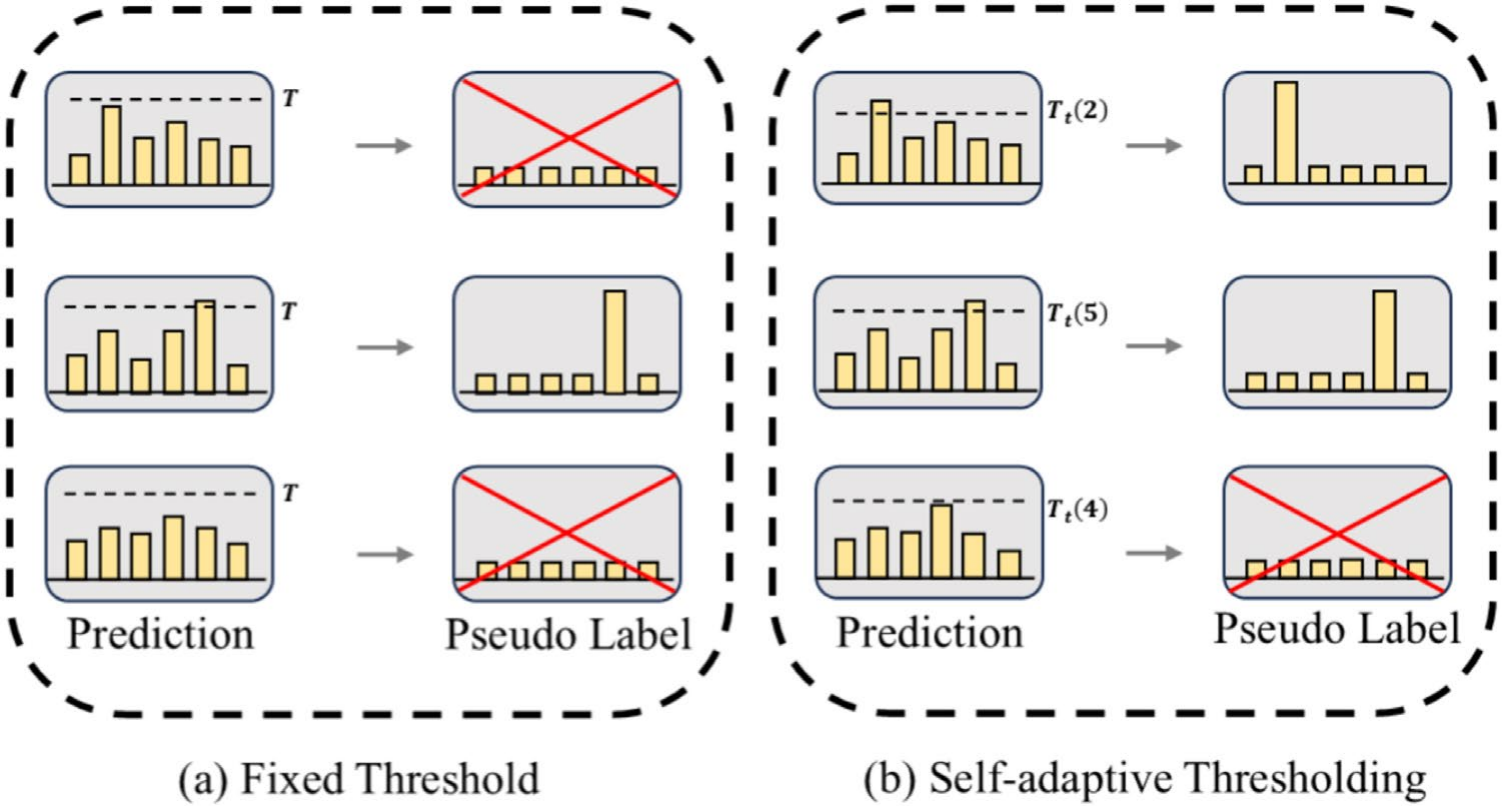
Comparison of two confidence threshold methods.

As shown in Fig. 5, the adaptive threshold improves pseudo-label utilization in early training and reduces false pseudo-label effects in late training compared to the fixed threshold.

Self-adaptive class fairness regularization encourages the model to make diverse predictions in all categories, which helps to generate diverse predictions in the early training phase and improves the performance of the model with fewer data. Self-adaptive class fairness regularization is achieved by adjusting the histogram distribution of the expected output probability, and the $L_f$ calculation of the loss of self-adaptive class fairness regularization is shown in Eq. (10):

10 $$\begin{aligned} L_f=-H\left( \frac{\left( \widetilde{\theta _t} / \widetilde{h_t}\right) }{\sum \left( \widetilde{\theta _t} / \widetilde{h_t}\right) }, \frac{({\bar{\theta }} / {\bar{h}})}{\sum ({\bar{\theta }} / {\bar{h}})}\right) \end{aligned}$$

where $H$ is the entropy function used to calculate the difference between the two probability distributions, $\widetilde{\theta _t}$ is the EMA of the predicted probabilities of the model for each category in the *t*-th iteration, $\widetilde{h_t}$ is the EMA of the histogram distribution in the *t*-th iteration, and ${\bar{\theta }}$ and ${\bar{h}}$ are the probability distributions being performed histogram normalization processing to $\widetilde{\theta _t}$ and $\widetilde{h_t}$, respectively.

The calculation $L_u$ of the loss of unsupervised learning training for the *t*-th iteration is shown in Eq. (11):

11 $$\begin{aligned} {L}_{u}=\left\{ \begin{array}{cc}\frac{1}{\mu B}\displaystyle \sum _{b=1}^{\mu B}H(\widehat{{q}_{b}},{Q}_{b}), & {T}_{p}\le {T}_{t} \\ \frac{1}{\mu B}\displaystyle \sum _{b=1}^{\mu B}\mathbbm {1}(max({q}_{b})>{T}_{t}(argmax({q}_{b})))\cdot H(\widehat{{q}_{b}},{Q}_{b}), & {T}_{p}> {T}_{t} \end{array}\right. \end{aligned}$$

where $q_b$ and $Q_b$ represent the prediction probability distributions of the model for weakly enhanced unlabeled samples and strongly enhanced unlabeled samples, respectively. $\widehat{{q}_{b}}$ represents the one-hot label converted from the $q_b$. $T_p$ represents the prediction probability of the unlabeled sample, $T_t$ represents the confidence threshold, and $\mathbbm {1}(\cdot )$ represents the indicator function.

### Total loss function

The total loss function of the proposed method is composed of supervised learning loss $L_s$, multi-source feature interaction constraint loss $L_c$, unsupervised learning loss $L_u$, and self-adaptive class fairness regularization loss $L_f$, which are calculated as shown in Eq. (12):

12 $$\begin{aligned} L={L}_{s}+{{w}_{c}}{L}_{c}+{{w}_{u}}{L}_{u}+{{w}_{f}}{L}_{f} \end{aligned}$$

where ${{w}}_{c}$, ${{w}}_{u}$ and ${{w}}_{f}$ are the corresponding weights of multi-source feature interaction constraint loss ${{L}}_{c}$, unsupervised learning loss ${{L}}_{u}$ and self-adaptive class fairness regularization loss ${{L}}_{f}$, respectively.

## Experiments

Considering the influence of the damping coefficient and the ’division by zero’ issue on the accuracy of working condition recognition, this paper selects measured ground dynamometer cards and measured electrical power cards (both are binary images) as the main information sources to analyze the working status of sucker-rod pumping wells comprehensively. The measured ground dynamometer card is plotted based on the displacement and load data of the polished rod. The measured electrical power card is drawn by the displacement of the polished rod and the effective electrical power of the motor (without relying on the specific feature parameter calculation through electrical signals, thus avoiding the ’division by zero’ issue).

The experimental working condition dataset is derived from a high-pressure, low-permeability thin oil reservoir block in an oilfield in China. The working condition samples used in the experiment are from 60 different oil wells over three consecutive calendar years of sucker-rod pumping well production in the above reservoir conditions, and are strictly selected according to the operation records of the oil wells. Each working condition sample is composed of data points collected at the actual corresponding acquisition time in the production site for both the measured ground dynamometer card and the measured electrical power card. The dataset includes 11 typical working conditions, with 150 samples for each condition, totaling 1, 650 samples. The 11 typical working conditions are normal, lack of supply liquid, pump leakage, tubing leakage, standing valve leakage, traveling valve leakage, assist-blowing, rod cutting, stuck pump, traveling valve failing, and wax precipitation.

This paper uses PyTorch 1.13.0 as the basic development framework, with the operating environment being Ubuntu 20.04, and NVIDIA RTX 3090 GPU. The training dataset and the test dataset contain 825 samples respectively, covering 11 working conditions, with 75 samples for each condition. All samples undergo identical preprocessing with color inversion and normalization. The experimental results are calculated as the averages of 10 runs. All comparative methods are evaluated under identical experimental conditions. The accuracy and F1-Score (Macro F1) are selected as the evaluation metrics.

To verify the effectiveness and practicability of the proposed method, this paper designs a series of experiments, which are divided into the following five parts: (a) Comparison of recognition results of different methods based on curvelet pooling and wavelet pooling; (b) Comparison of recognition results of different methods based on single-source and multi-source; (c) Comparison of different multi-source semi-supervised condition recognition methods under different labeled sample ratios; (d) Sensitivity analysis of key hyperparameters, analyzing the influence of key hyperparameters on model performance and determining the optimal parameter configuration; (e) Ablation experiments, evaluating the contribution of key components in the proposed method to model performance and verifying the rationality of the method design.

### Comparison of recognition results of different methods based on curvelet pooling and wavelet pooling

To verify the superiority of curvelet pooling implemented based on USFFT in supervised scenarios, different types of wavelet pooling are introduced into recognition methods based on single-source(S-ResNet) and attention mechanism-based multi-source feature fusion extraction(AMMFFE-ResNet), and then compared with the proposed method in this paper (AMMFFE-CP-ResNet-50). Specifically, these include single-source working condition recognition methods optimized by wavelet pooling based on different basis functions (S-WP-db4-ResNet-50, S-WP-bior3.3-ResNet-50), lifting wavelet pooling (S-LWP-db4-ResNet-50, S-LWP-bior3.3-ResNet-50), dual-tree complex wavelet pooling (S-DTCWP-ResNet-50), and USFFT-based curvelet pooling (S-CP-ResNet-50), and attention mechanism-based multi-source feature fusion extraction working condition recognition methods optimized by wavelet pooling based on different basis functions (AMMFFE-WP-db4-ResNet-50, AMMFFE-WP-bior3.3-ResNet-50), lifting wavelet pooling (AMMFFE-LWP-db4-ResNet-50, AMMFFE-LWP-bior3.3-ResNet-50), and dual-tree complex wavelet pooling (AMMFFE-DTCWP-ResNet-50). The experimental results are shown in Table 2.

**Table 2:** Comparison of recognition results based on different pooling methods.

Working condition recognition methods	Measured dynamometer card		Measured electrical power card		Measured dynamometer card and measured electrical power card	
	Accuracy (%)	F1-score	Accuracy (%)	F1-score	Accuracy (%)	F1-score
Different pooling methods under single-source						
S-WP-db4-ResNet-50	98.81	0.9879	98.32	0.9828	–	–
S-WP-bior3.3-ResNet-50	98.87	0.9883	98.36	0.9834	–	–
S-LWP-db4-ResNet-50	98.70	0.9868	98.39	0.9837	–	–
S-LWP-bior3.3-ResNet-50	98.92	0.9889	98.44	0.9839	–	–
S-DTCWP-ResNet-50	98.87	0.9886	98.32	0.9829	–	–
S-CP-ResNet50	**99.16**	**0.9915**	**98.47**	**0.9839**	–	–
Different pooling methods under multi-source						
AMMFFE-WP-db4-ResNet-50	–	–	–	–	99.36	0.9935
AMMFFE-WP-bior3.3-ResNet-50	–	–	–	–	99.24	0.9924
AMMFFE-LWP-db4-ResNet-50	–	–	–	–	99.19	0.9918
AMMFFE-LWP-bior3.3-ResNet-50	–	–	–	–	99.39	0.9937
AMMFFE-DTCWP-ResNet-50	–	–	–	–	98.97	0.9897
AMMFFE-CP-ResNet-50 (ours)	–	–	–	–	**99.52**	**0.9952**

Significant values are in bold

As can be seen from Table 2: (a) In the pooling layer optimization experiments based on single-source and multi-source, the optimization effect of curvelet pooling is better than that of wavelet pooling. (b) Compared with the optimal multi-source working condition recognition method optimized by basis wavelet pooling, the accuracy and F1-Score of the proposed method are improved by 0.16% and 0.0017, respectively; compared with the optimal multi-source working condition recognition method optimized by lifting wavelet pooling, the accuracy and F1-Score are improved by 0.13% and 0.0015, respectively. (c) Compared with the multi-source working condition recognition method optimized by dual-tree complex wavelet pooling, the accuracy and F1-Score of the proposed method are improved by 0.55% and 0.0055, respectively. In supervised scenarios, although the improvement in accuracy of our method is modest, the enhancement in F1-Score further demonstrates the robustness of the performance improvement achieved by the proposed method. Through the above analysis, the curvelet pooling can effectively mitigate the problem that the inherent pooling design of traditional CNN affects the model recognition performance.

### Comparison of recognition results of different methods based on single-source and multi-source

To verify the effectiveness and superiority of the proposed attention mechanism-based multi-source fusion feature extraction method in supervised scenarios, ResNet series models and ViT (Vision Transformer)^42^ models are selected for experiments. The experimental methods include single-source working condition recognition methods (SResNet-18/34/50/101/152, SViT), attention mechanism-based multi-source fusion feature extraction working condition recognition methods (AMMFFEResNet-18/34/50/101/152, AMMFFE-LWP-bior3.3-ResNet-50, AMMFFEViT, AMMFFE-ResNet-50_SE, AMMFFE-ResNet-50_SELF, and the proposed method AMMFFE-CP-ResNet-50). In addition, the traditional multi-source feature connection-based working condition recognition method (MResNet-50), the interpretable multi-view graph convolutional neural network (IMvGCN)^43^, and two semi-supervised recognition methods based on attention mechanism-based multi-source fusion feature extraction ( AMMFFE-InfoMatch-ResNet-50^44^, AMMFFE-SequenceMatch-ResNet-50^45^) are introduced as comparisons. The experimental results are shown in Table 3.

**Table 3:** Comparison of recognition results based on single-source and multi-source methods.

Working condition recognition methods	Measured dynamometer card		Measured electrical power card		Measured dynamometer card and Measured electrical power card	
	Accuracy (%)	F1-score	Accuracy (%)	F1-score	Accuracy (%)	F1-score
Single-source ResNet series models						
SResNet-18	98.66	0.9863	98.02	0.9788	–	–
SResnet-34	98.23	0.9821	97.75	0.9769	–	–
SResnet-50	**98.79**	**0.9876**	**98.22**	**0.9818**	–	–
SResnet-101	98.43	0.9838	98.05	0.9802	–	–
SResnet-152	98.33	0.9829	97.27	0.9724	–	–
Single-source other models						
SViT	98.48	0.9847	97.13	0.9712	–	–
Multi-source different working condition recognition methods						
AMMFFEResNet-18	–	–	–	–	99.10	0.9907
AMMFFEResNet-34	–	–	–	–	99.03	0.9901
AMMFFEResNet-50	–	–	–	–	99.37	0.9936
AMMFFEResNet-101	–	–	–	–	99.13	0.9911
AMMFFEResNet-152	–	–	–	–	98.84	0.9879
AMMFFEViT	–	–	–	–	99.24	0.9922
MResNet-50	–	–	–	–	99.32	0.9930
AMMFFE-ResNet-50_SE	–	–	–	–	98.92	0.9891
AMMFFE-ResNet-50_SELF	–	–	–	–	98.86	0.9884
AMMFFE-LWP-bior3.3-ResNet-50	–	–	–	–	99.39	0.9937
IMvGCN	–	–	–	–	96.57	0.9656
AMMFFE-InfoMatch-ResNet-50	–	–	–	–	97.63	0.9764
AMMFFE-SequenceMatch-ResNet-50	–	–	–	–	99.26	0.9926
AMMFFE-CP-ResNet-50 (ours)	–	–	–	–	**99.52**	**0.9952**

Significant values are in bold

From Table 3, it can be seen that: (a) Among the single-source and multi-source feature attention fusion methods without pooling improvements, AMMFFEResNet-50 achieves the best recognition performance, verifying the effectiveness of the proposed attention mechanism-based multi-source fusion feature extraction. (b) The proposed AMMFFE-CP-ResNet-50 outperforms the optimal wavelet pooling method, further validating the effectiveness of curvelet pooling in optimizing CNNs for collaborative learning of low-frequency and high-frequency information. (c) Under the premise of multi-source feature interaction via multi-head self-attention, the adaptive attention-based fusion method, AMMFFEResNet-50 improves accuracy and F1-Score by 0.45% and 0.0045 compared to the SE attention mechanism, and by 0.51% and 0.0052 compared to the self-attention mechanism, and it is worth noting that in supervised scenarios, the consistent improvements in both accuracy and F1-Score further demonstrate the robustness of the performance enhancement attained by the proposed method, confirming the superiority of adaptive attention-based fusion.

### Comparison of different multi-source semi-supervised condition recognition methods under different labeled sample ratios

**Fig. 6:**
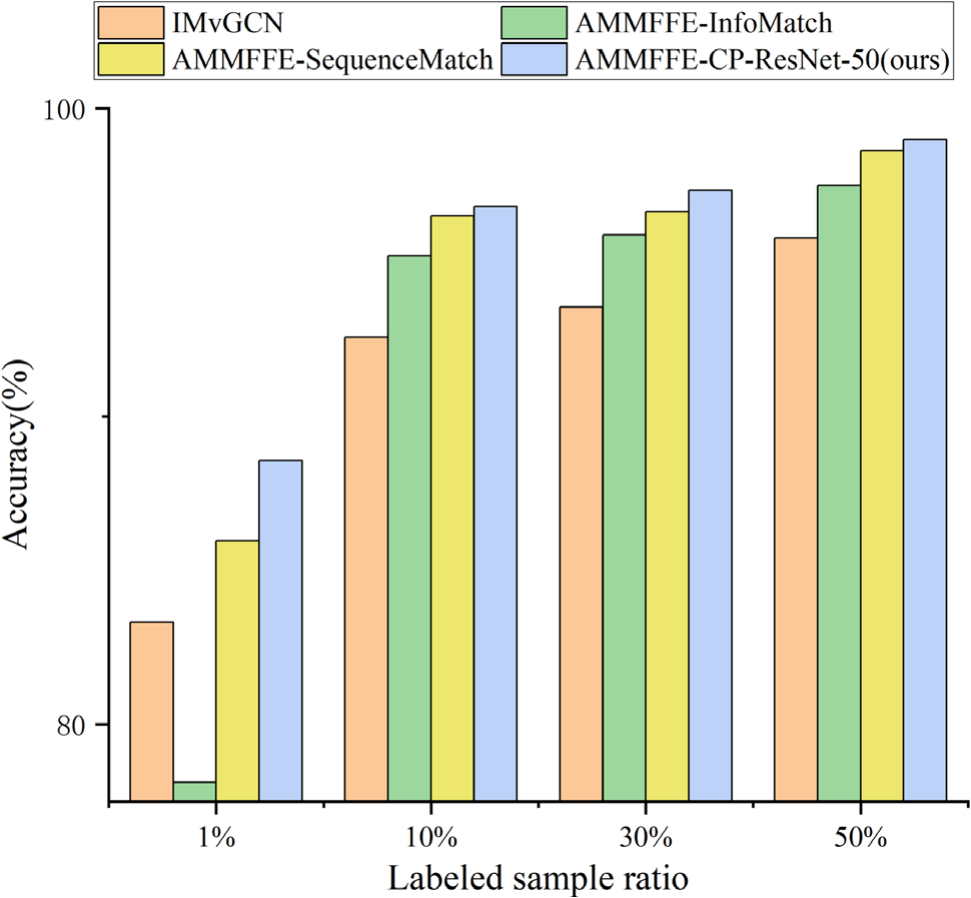
Comparison of the recognition effects of different semi-supervised learning methods.

To validate the proposed method’s superiority in semi-supervised scenarios, multi-source working condition recognition results under different semi-supervised ratios (1%, 10%, 30%, 50%, corresponding to labeled samples n=11, n=77, n=242, n=407) are compared based on the first set and the second set of experiments. For all settings, the splits of labeled and unlabeled samples are kept consistent across different sources to ensure fair comparisons. The results are shown in Table 4, with comparisons to different semi-supervised learning methods illustrated in Fig. 6.

**Table 4:** Comparison of different multi-source semi-supervised condition recognition methods under different labeled sample ratios.

Working condition recognition methods	Labeled sample ratio				
		1%	10%	30%	50%
MResNet-50	Accuracy (%)	85.52	95.95	96.67	98.80
	F1-Score	0.8521	0.9588	0.9664	0.9878
AMMFFE-ResNet-50	Accuracy (%)	86.67	96.07	96.90	98.81
	F1-Score	0.8637	0.9605	0.9687	0.9880
AMMFFE-WP-db4-ResNet-50	Accuracy (%)	85.94	96.10	96.74	98.90
	F1-Score	0.8599	0.9603	0.9670	0.9887
AMMFFE-WP-bior3.3-ResNet-50	Accuracy (%)	85.73	96.10	96.78	98.92
	F1-Score	0.8551	0.9605	0.9679	0.9891
AMMFFE-LWP-db4-ResNet-50	Accuracy (%)	85.56	95.82	96.44	98.51
	F1-Score	0.8526	0.9578	0.9638	0.9848
AMMFFE-LWP-bior3.3-ResNet-50	Accuracy (%)	86.85	96.34	97.20	98.86
	F1-Score	0.8649	0.9632	0.9719	0.9886
IMvGCN	Accuracy (%)	83.31	92.56	93.55	95.78
	F1-Score	0.8331	0.9256	0.9355	0.9579
AMMFFE-InfoMatch-ResNet-50	Accuracy (%)	78.12	95.21	95.88	97.49
	F1-Score	0.7586	0.9524	0.9601	0.9748
AMMFFE-SequenceMatch-ResNet-50	Accuracy (%)	85.96	96.51	96.64	98.62
	F1-Score	0.8420	0.9649	0.9670	0.9860
AMMFFE-CP-ResNet-50 (ours)	Accuracy (%)	**88.57**	**96.80**	**97.34**	**98.98**
	F1-Score	**0.8819**	**0.9669**	**0.9732**	**0.9897**

Significant values are in bold

From Table 4, it can be seen that: (a) The proposed AMMFFE-CP-ResNet-50 outperforms state-of-the-art semi-supervised methods in multi-source condition recognition.Specifically, compared with IMvGCN, at 1% labeled sample ratio, (accuracy/F1-Score +5.26%/0.0448); at 10% (accuracy/F1-Score +4.24%/0.0413); at 30% (accuracy/F1-Score +3.79%/0.0377); and at 50% (accuracy/F1-Score +3.20%/0.0318). Compared with AMMFFE-InfoMatch, at 1% (accuracy/F1-Score +10.45%/0.1233); at 10% (accuracy/F1-Score +1.59%/0.0145); at 30% (accuracy/F1-Score +1.46%/0.0131); and at 50% (accuracy/F1-Score +1.49%/0.0149). Compared with AMMFFE-SequenceMatch, at 1% (accuracy/F1-Score +2.61%/0.0339); at 10% (accuracy/F1-Score +0.29%/0.002); at 30% (accuracy/F1-Score +0.7%/0.0062); and at 50% (accuracy/F1-Score +0.36%/0.0037). (b) AMMFFE-ResNet-50 outperforms MResNet-50 under all semi-supervised ratios, particularly at 1% (accuracy and F1-Score improved by 1.15% and 0.0116), verifying the effectiveness of multi-source feature interaction and fusion in semi-supervised scenarios. (c) The proposed AMMFFE-CP-ResNet-50 surpasses the optimal pooling method (AMMFFE-LWP-bior3.3-ResNet-50) under all ratios, especially at 1% (accuracy/F1-Score +1.72%/0.017) and 10% (accuracy/F1-Score +0.46%/0.0037), confirming the superiority of curvelet pooling-based CNN optimization in semi-supervised settings.

### Sensitivity analysis of key hyperparameters

**Fig. 7:**
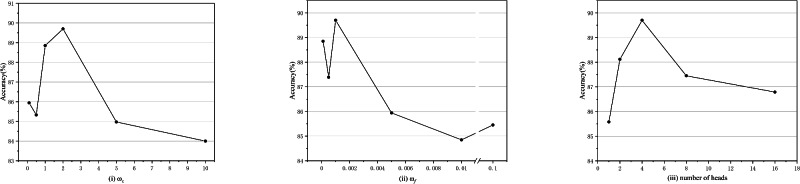
Sensitivity analysis of key parameters in the proposed method under 1% labeled samples.

To determine optimal configurations, sensitivity analysis is conducted on key hyperparameters (multi-source feature interaction constraint loss weight $w_c$ (Fig. 7(i)), self-adaptive class fairness regularization weight $w_f$ (Fig. 7(ii)), and number of multi-head self-attention heads (Fig. 7(iii)) in the Transformer encoder) at a 1% labeled sample ratio. The tuning ranges are $w_c \in [0.1, 0.5, 1, 2, 5, 10]$, $w_f \in [0.0001, 0.0005, 0.001, 0.005, 0.01, 0.1]$, and heads $\in [1, 2, 4, 8, 16]$.

As shown in Fig. 7: (a) The analysis results for $\omega _c$ and $\omega _f$ exhibit similar patterns, with accuracy showing a distinct nonlinear trend: initially increasing to a peak value before declining. The peak accuracy is achieved at $\omega _c = 2$ and $\omega _f = 0.001$, respectively. (b) $\omega _c$ and $\omega _f$ serve as constraints for different loss terms in the objective function. Insufficient weighting may lead to inadequate constraint, while excessive weighting may restrict model learning capacity. Both scenarios can impair the model’s fitting ability. Appropriate weighting balances regularization and loss functions, thereby optimizing model performance. (c) Accuracy demonstrates a clear inverted-U trend as the number of attention heads increases, first rising and then declining. The model achieves peak accuracy with 4 attention heads, indicating optimal multi-source feature interaction learning. In summary, based on the experimental results of the sensitivity analysis, our key hyperparameters are set as follows: $\omega _c$ = 2, $\omega _f$ = 0.001, and the number of heads in the multi-head self-attention mechanism is 4.

### Analysis of model inference time

In consideration of computational cost, inference efficiency is an important factor for the practical deployment of deep learning models. Therefore, we evaluate the inference time of the proposed model using different pooling strategies, and the results are presented in Table 5. The reported values are averaged over 100 runs to ensure reliability, and the evaluation is conducted under the setting with 1% labeled sample ratio.

**Table 5:** Average inference time per sample under different pooling operations.

Model	Inference time (ms)
AMMFFE-ResNet-50 (max-pooling)	20.3
AMMFFE-CP-ResNet-50 (ours)	36.2

Although the proposed curvelet pooling increases the inference time compared with max-pooling, this additional cost mainly stems from the multi-scale and directional transforms inherent in curvelet pooling. However, comprehensively considering the important actual oil extraction production indicators, such as enhancing the recovery rate of oil wells, the further improvement of the recognition accuracy of oil well working conditions is far more important than the increase in its computational cost.

### Ablation study

Ablation tests at 1% labeled samples verify key module contributions:

- Comparing the method without multi-source feature interaction (AMMFFE-CP-ResNet-50(w/o FI)) to the proposed method.
- Replacing the adaptive attention-based fusion with average weight/fixed weight strategies (AMMFFE-CP-ResNet-50(w/FI-AVE), AMMFFE-CP-ResNet-50(w/FI-0.5FIX)).

**Figure 8:**
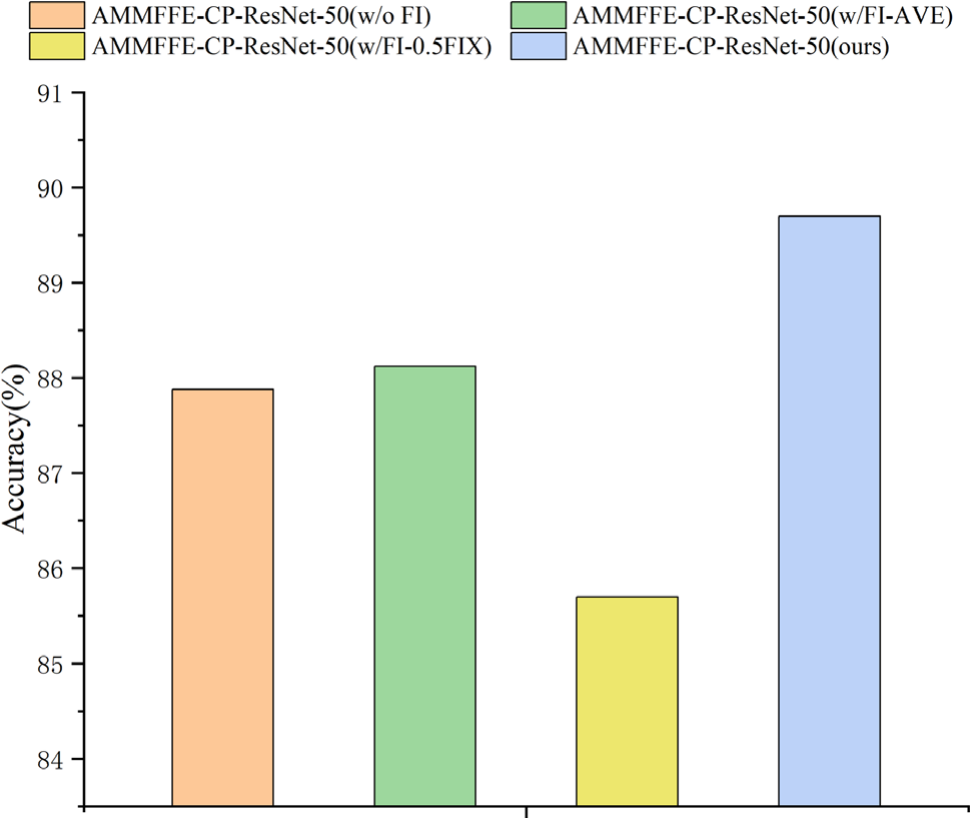
Comparison of the recognition effects of different semi-supervised learning methods.

Experimental results are presented in Table 6 and Fig. 8.

**Table 6:** Ablation experiments with 1% labeled samples.

Working condition recognition methods	Accuracy (%)
AMMFFE-CP-ResNet-50 (w/o FI)	87.88
AMMFFE-CP-ResNet-50 (w/ FI-AVE)	88.12
AMMFFE-CP-ResNet-50 (w/ FI-0.5FIX)	85.70
AMMFFE-CP-ResNet-50 (ours)	**89.70**

Significant values are in bold

As shown in Table 6 and Fig. 8: (a) AMMFFE-CP-ResNet-50(w/o FI) shows 1.82% lower accuracy, confirming that multi-head self-attention-driven feature interaction enhances cross-source information learning. (b) AMMFFE-CP-ResNet-50(w/ FI-AVE) and AMMFFE-CP-ResNet-50(w/ FI-0.5FIX) exhibit 1.58% and 4% accuracy drop respectively, validating that adaptive attention-based fusion improves global feature representation and recognition performance of the model.

**Table 7:** Model performance with different number of training samples per class.

Number of training samples per class	Accuracy (%)
30	85.45
50	88.18
75 (ours)	**89.70**

Significant values are in bold

In addition to the key module ablation, we conducted a sample size ablation experiment to evaluate the robustness of the proposed method under reduced training samples. Specifically, for each class, the number of labeled samples was randomly reduced to 50 and 30, and the model performance was recorded. The results are presented in Table 7. As the training sample size decreases, AMMFFE-CP-ResNet-50 maintains comparatively good performance across different scales, indicating its robustness with limited training data.

## Conclusion and future work

This paper proposes a working condition recognition method featuring three key characteristics: curvelet pooling, attention mechanism-based measured multi-source fusion feature extraction, and multi-source semi-supervised classification deep learning. Specifically, the measured ground dynamometer cards and measured electrical power cards are selected as major information sources for working condition recognition. The curvelet pooling layer, built upon the second-generation curvelet transform, is introduced to replace the max-pooling layer in ResNet-50, which helps alleviate certain limitations of conventional convolutional neural networks in feature representation. Further, an attention-based multi-source fusion feature extraction framework is designed to enable feature interaction through multi-head self-attention and achieve feature fusion via an adaptive attention mechanism. On this basis, the multi-source fused features are processed with a semi-supervised classification algorithm that incorporates a pseudo-label confidence dynamic adjustment strategy and adaptive class fairness regularization, forming an integrated approach for multi-source semi-supervised working condition recognition. Extensive experiments validate the effectiveness of the proposed method.

However, this research also has certain limitations: (a) Although the proposed multi-source semi-supervised learning method has shown effectiveness, its performance under extremely few labeled samples requires further improvement, which may not be sufficient to fully meet the demands of real-world industrial applications; (b) The research primarily focuses on theoretical aspects and improving recognition performance, without considering the computational and memory constraints of embedded devices, which may limit the immediate applicability of the method in resource-constrained industrial environments.

In order to improve the technical level and application effect in the field of oil extraction production, the following directions can be further explored: (a) Considering the lack of a general framework in this field that simultaneously addresses multiple practical challenges, future work will aim to enhance the generalization of the proposed method across different oil reservoir geologic condition. This would involve integrating techniques such as imbalanced learning^46^ to handle skewed data distributions, incremental learning^47^ to continuously incorporate new data without retraining, domain adaptation^48^ to mitigate distribution shifts between different operating site, etc., ultimately building a more robust and versatile working condition recognition system. (b) Focus on the improvement of semi-supervised learning algorithms for working condition recognition in sucker-rod pumping wells, such as enhancing the quality of pseudo-labels to improve the model performance with very few labeled samples. (c) Explore the lightweight deployment strategies of the proposed multi-source semi-supervised deep learning model on edge devices of sucker-rod pumping wells, such as curvelet transform optimization technology, deep model quantization compression technology, to further improve the practicability and promotion value of the working condition recognition system for sucker-rod pumping wells in complex oil extraction production sites, and provide efficient and stable edge intelligent solutions for intelligent oilfield construction.

## Data Availability

The datasets used and/or analysed during the current study available from the corresponding author on reasonable request.
